# Advanced Microrobots Driven by Acoustic and Magnetic Fields for Biomedical Applications

**DOI:** 10.34133/cbsystems.0386

**Published:** 2025-11-10

**Authors:** Tingting Wang, Zhuo Chen, Qiang Huang, Tatsuo Arai, Xiaoming Liu

**Affiliations:** ^1^ Key Laboratory of Biomimetic Robots and Systems, Ministry of Education, State Key Laboratory of Intelligent Control and Decision of Complex System, and School of Mechatronical Engineering, Beijing Institute of Technology, Beijing 100081, China.; ^2^ Center for Neuroscience and Biomedical Engineering, The University of Electro-Communications, Tokyo 1828585, Japan.

## Abstract

Microrobots driven by magnetic and acoustic fields have shown great potential in multiple biomedical applications due to their excellent biocompatibility, wireless actuation, access to confined environments, and tissue penetration. A single physical actuation method often meets inevitable limitations and complications, such as the limited propulsion of the magnetic actuation and difficult direction control of the acoustic actuation. This review summarizes the current progress of hybrid magneto-acoustic actuation to address the limitations of single magnetic or acoustic actuation. First, we review the research on microrobots driven by single magnetic and acoustic fields and clarify the properties of each physical actuation. Then, we summarize 2 forms of hybrid magnetic-acoustic actuation: (a) magnetic steering and acoustic propulsion and (b) magnetic propulsion and acoustic manipulation. The state-of-the-art applications of magneto-acoustic microrobots, including targeted drug delivery, minimally invasive surgery, and medical imaging, are presented to demonstrate their great potential in biology and clinics. This article finally discusses current challenges and potential developments in magneto-acoustic robotics to provide a reliable path for designing and applying hybrid magneto-acoustic actuation methods.

## Introduction

**Fig. 1.**
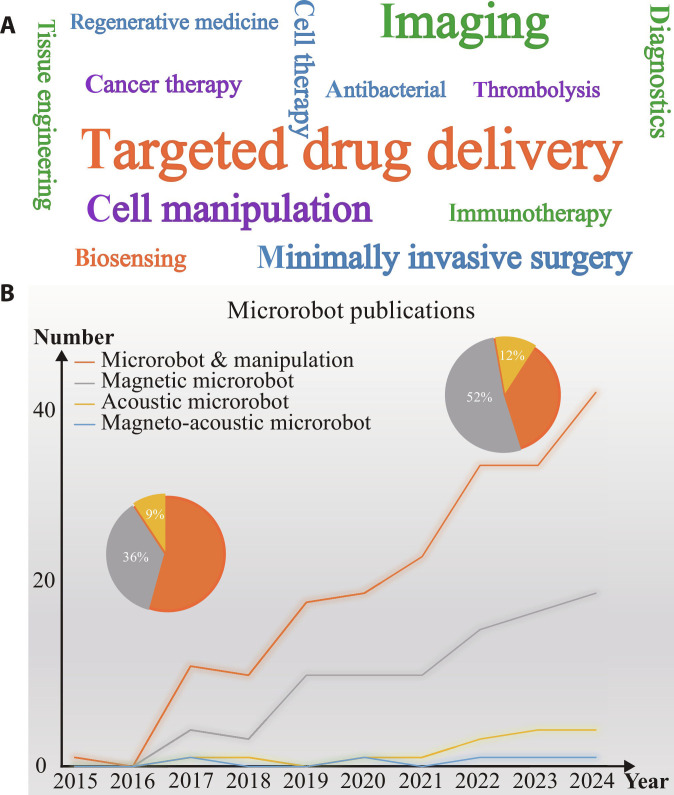
Microrobot publications in *Science*, *Nature*, and their sub-journals such as *Science Advances*, *Science Robotics*, and *Nature Communications* from 2015 to 2024. (A) The word cloud illustrates the most frequent applications of microrobots in the biomedical field. The font size positively correlates with the number of relevant publications on the Web of Science (WoS). (B) The number of publications featuring the keywords “microrobots”, “manipulation”, “acoustic microrobot /actuator/swimmer”, “magnetic microrobot /actuator/swimmer”, and “magnetic and acoustic microrobot/actuator/swimmer” within the period from 2015 to 2024, according to the WoS.

Microrobots, defined as miniaturized systems spanning dimensions from several nanometers to submillimeters, represent a cutting-edge class of devices engineered to navigate through biological fluids and tissues and localize at specific targets. These systems have emerged as versatile platforms for advancing microscopic investigations and executing high-precision manipulations at the microscale, bridging the gap between nanoscale interactions and macroscopic functionality [1]. Recently, microrobots have shown remarkable progress with advances in micro/nanofabrication, materials, and actuation mechanisms [2–7]. Microrobots exhibit the capability of carrying out operations at the microscopic level due to their miniaturized dimensions, untethered actuation, and multiple locomotion. They can access deep, complex, and narrow regions of the human body with minimal invasiveness, such as blood vessels and brain tissue. Consequently, microrobots hold extensive potential for biomedical applications [8]. We evaluated the utilization frequency of microrobots within distinct biomedical fields in Fig. 1A, which shows their widespread applications, particularly in targeted drug delivery [9,10], minimally invasive surgery [11], cell manipulation [12], and imaging [13]. Moreover, statistics on microrobot research works published in high-impact journals indicate that magnetically driven microrobots are mainstreaming, while acoustic and magneto-acoustic microrobots hold great potential (Fig. 1B).

Microrobots face challenges of propulsion and motion control in low Reynolds number fluid environments, where viscous force dominates over inertial force. Thus, effective actuation mechanisms for microrobots have been proposed and categorized by external energy fields into magnetic [14–16], electric [17–19], acoustic [20–22], and optical [23–25] approaches. Based on the interaction between an electric field and conductive materials, electric field actuation can generate mechanical force. However, applying a strong electric field in biological systems is constrained by potential cellular damage, rendering this approach unsuitable for in vivo environments [17]. Optical field actuation, which utilizes optical gradient or photophoretic forces, offers high manipulation precision and flexibility but suffers from limited tissue penetration, particularly in deep tissues, due to poor light transmission [26].

In contrast, magnetic and acoustic field actuation methods have gained increasing attention in biomedical applications due to their favorable biocompatibility and superior tissue penetration capabilities [20,27,28]. According to the Web of Science database, the number of publications containing keywords such as “microrobot” or “micromanipulation”, “magnetic microrobot”, “acoustic microrobot”, and “magnetic and acoustic microrobot”, published in high-impact journals like *Nature*, *Science*, and their sub-journals, over the past decade is shown in Fig. 1B. This indicates that with the development of microrobots, magnetic microrobots have consistently attracted substantial attention, while acoustic microrobots are gradually emerging as a powerful branch. Meanwhile, magneto-acoustic microrobots are gaining increasing attention.

As a versatile and widely adopted method, magnetic actuation offers excellent biocompatibility, untethered maneuverability, and precise control, driving substantial interest in its application [28,29]. Low-intensity magnetic fields can penetrate biological tissues, enabling contactless manipulation in vivo. Clinical studies have further confirmed the safety and nontoxic nature of this approach for human use [30]. In addition, the rapid development of diverse magnetic control platforms has greatly enhanced the degrees of freedom in locomotion, thereby improving the controllability and functional complexity of magnetic microrobots [31]. However, the rapid spatial decay of magnetic fields inherently limits their actuation force, requiring high input power to enable effective remote manipulation. Furthermore, many magnetically actuated microrobots depend on specific deformation modes to perform complex operations. This operational strategy increases fabrication complexity and limits the applicability of magnetic microrobots in complex tasks.

Acoustic actuation has been recognized as a highly safe and efficient driving method. Ultrasound has been widely used in clinical imaging, demonstrating excellent biocompatibility and in vivo safety [32]. Acoustic waves are capable of penetrating deep biological tissues and can generate strong propulsion for microrobots with relatively low power consumption [33]. In addition, acoustically induced physical effects, such as cavitation [34] and sonochemical reactions [35], can be harnessed to enable specific functionalities, including drug release. However, achieving precise directional control of microrobot movement remains difficult, which constrains high-accuracy navigation in complex biological environments [36].

Precision trajectory and velocity control of microrobots ​remain fundamental requirements for clinical implementations, but ​no existing actuation paradigm achieves comprehensive performance benchmarks across regulatory accuracy and operational versatility. Hybrid magneto-acoustic actuation strategies ​are emerging as a systematic solution to address these limitations. By combining the precision of magnetic control with the substantial propulsion force and functional activation provided by the acoustic field, this hybrid approach promises to overcome the shortcomings of individual methods. Such integration holds great potential for advancing applications in targeted drug delivery, minimally invasive surgery, and other biomedical scenarios that require multimodal control within complex physiological environments.

Advanced progress in the fabrication, actuation, and application of magnetically and acoustically driven microrobots has been reported [27,31,37–39]. However, no comprehensive review has specifically focused on hybrid magneto-acoustic microrobots. This review aims to fill this gap by providing a systematic overview of this emerging field. In this review, recent advances in the research and development of hybrid magneto-acoustic microrobots are discussed. First, the actuation mechanisms are categorized based on their working principles. Then, the key applications of hybrid magneto-acoustic microrobots are systematically summarized. Finally, the current challenges and potential future developments in this field are provided.

## Actuation Mechanisms of Acoustic and Magnetic Microrobot

### Magnetic actuation

Magnetic actuation is a popular method for propelling and controlling microrobots applied in the biomedical field due to the advantages of good penetration, remote driving, and biocompatibility. Magnetically actuated microrobots move within a magnetic field due to the influence of either force or torque [40]. Therefore, the following discussion will explore their working mechanisms from the perspective of these 2 physical principles.

#### Magnetic torque

**Fig. 2.**
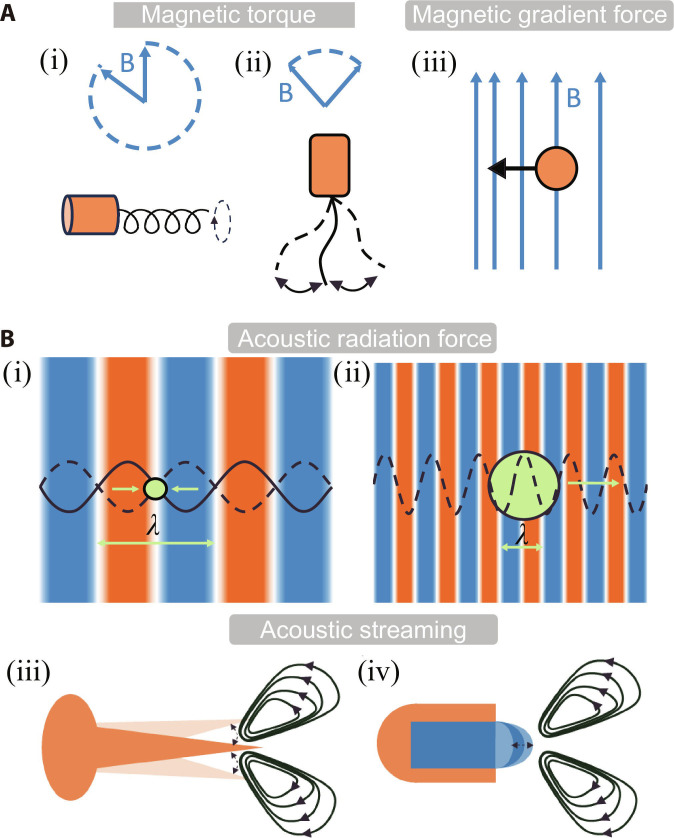
The actuation mechanisms of magnetic and acoustic microrobots. (A) The actuation mechanisms of magnetic microrobots include 3 distinct modes: propulsion in (i) a rotating magnetic field, (ii) an oscillating magnetic field, and (iii) a gradient magnetic field. (B) The actuation mechanisms of acoustic microrobots include 2 distinct modes: acoustic radiation forces acting on (i) small agents (ka≪1) under standing waves and (ii) larger agents (ka > 1) under traveling wave propagation, and acoustic streaming effects around (iii) the sharp edge and (iv) the oscillating bubble under acoustic excitation.

The magnetic torque exerted on a target object can be expressed as τ=m·B, where m is the magnetic moment and B is the external magnetic field. When applying a uniform magnetic field, the magnetic microrobot rotates under the effect of the magnetic moment until the magnetic moment is aligned with the magnetic field. Therefore, to enable continuous propulsion of the robot in a uniform magnetic field, the field must vary over time, such as a rotating or oscillating magnetic field [41]. The geometric design of the microrobots driven by the rotating and oscillating magnetic fields plays a crucial role in determining motion performance. Microrobots with helical or spherical structures are commonly used under rotating magnetic fields. Helical microrobots rotate around the axis of rotation, generating a net displacement along this axis to achieve forward motion, while spherical microrobots propel themselves through a self-rolling mode under the rotating magnetic field. Inspired by the swimming mechanisms of fish and flagellated microorganisms, microrobots driven by oscillating magnetic fields often feature flexible tails. In an oscillating magnetic field, the tail of the magnetic microrobot oscillates under the action of time-varying magnetic torque, and the microrobot is propelled by the undulatory motion (Fig. 2A).

Honda et al. [42] first proposed the actuation of a helical microrobot in a rotating magnetic field, which was composed of a small magnet and a helical wire. Nelson and colleagues have conducted a series of studies on magnetic helical microrobots, achieving precise control for swimming direction, speed [43], and swarm behavior [44,45] through structural design and magnetic field modulation. The helical microrobots have been practically implemented using biocompatible, degradable hydrogel materials [46] and advanced fabrication techniques such as 3-dimensional (3D) printing and 2-photon lithography. Accordingly, soft helical robots for biomedical applications were developed, including targeted drug delivery [47], single-cell manipulation [48], and therapeutic interventions in neurological disorders [49]. Alapan et al. [50] proposed a spherical microroller composed of magnetic Janus microparticles. Under a rotating magnetic field, the microroller rotates due to the influence of the magnetic torque, while the presence of asymmetric boundary hydrodynamic forces in a physiological flow environment converts this rotation into rolling along the surface. Dreyfus et al. [51] were the first to propose the propulsion of the magnetic microrobot in an oscillating magnetic field by using flexible artificial flagella, which is a chain-like assembly of superparamagnetic beads interconnected by DNA fibers. The asymmetric deformation of flagella enables the microrobot to move forward.

#### Magnetic gradient force

The magnetic gradient force acting on an object can be expressed as $F=\nabla \left(m\cdot B\right)$, where m is the magnetic moment and B is the external magnetic field. The magnetic gradient force drives the magnetic targets toward regions of increasing field intensity in a nonuniform magnetic field [52] (Fig. 2A). Compared to rotating and oscillating magnetic fields, gradient magnetic fields can manipulate microrobots of arbitrary shapes, eliminating the need for structural optimization. Li et al. [53] developed a burr-like porous spherical microrobot fabricated via 3D laser lithography, capable of navigating in different fluid environments under magnetic gradient field actuation. This microrobot achieved targeted cell transport and on-demand release in vivo. Xin et al. [54] developed environmentally adaptive shape-morphing microrobots by 4D laser printing. These hydrogel-based magnetic microrobots, designed with biomimetic crab-like and fish-like morphologies, execute complex micromanipulation tasks (grasping, transporting, and releasing microparticles) through the actuation and navigation of a magnetic gradient field. Beyond individual microrobot control, magnetic gradient fields enable programmable swarm manipulation. Nelson and colleagues [55] developed intravascular navigation of magnetic microswarms actuated by gradient forces generated through the permanent magnet. These swarms achieve upstream/downstream navigation in dynamic blood flow, enabling precise spatiotemporal targeting within the vessels. Integrated with ultrasound Doppler imaging, this strategy provides real-time tracking and spatiotemporal guidance of microswarms under physiological flow conditions.

Despite substantial advances in material engineering, propulsion mechanisms, and fabrication technologies [56], magnetically actuated microrobots remain challenged by 2 critical limitations. First, this operation relies on high-intensity magnetic fields or proximity-based actuation due to the rapid decay of magnetic intensity with increasing distance, resulting in excessive energy consumption and restricted operational workspace [29]. Second, current magnetic control strategies lack the capability for selective individual actuation within complex biological environments [10].

### Acoustic actuation

The acoustic field has emerged as another compelling actuation method for microrobots, offering a robust remote power source to propel devices deep within biological tissues. The physical effects of the acoustic field primarily manifest as acoustic radiation force and acoustic streaming. The following sections will elaborate on these 2 actuation mechanisms and the key design considerations for corresponding microrobots.

#### Acoustic radiation force

Acoustic radiation force arises when acoustic waves propagate through a fluid medium containing a target object due to the difference in acoustic properties (e.g., density and compressibility) between the fluid and the object. The acoustic pressure gradient generates a net mechanical force capable of displacing, trapping, or manipulating the target in traveling or standing wave fields [57]. The acoustic radiation force is derived from the first-order acoustic pressure acting on a target, comprising contributions from the gradient force and the scattering force [58]. When the target size is much smaller than the acoustic wavelength (ka≪1,k=2π∕λ, λ is the wavelength of the acoustic wave), the radiation force is predominantly governed by the gradient force in the acoustic field, which pushes the target toward pressure nodes or antinodes in the standing wave field [20,59]. In contrast, for a larger target (ka>1), the scattering force dominates and can drive the translational motion of the target in a traveling wave field [58,60,61] (Fig. 2B).

In both traveling and standing wave acoustic fields, microrobots propelled by acoustic radiation forces have been proposed for controlled motion. Standing wave fields enable the trapping and transporting of microrobots at nodal positions, while traveling wave fields facilitate guided navigation along the sound wave propagation direction. These approaches leverage acoustic radiation forces to achieve noncontact, programmable control of microrobots, expanding their potential for biomedical applications. Wang et al. [62] first reported the directional motion and rotation of asymmetrically dense nanorods in the acoustic field while confined in a standing wave nodal plane. Ahmed and colleagues [63] demonstrated microbubble manipulation within a zebrafish embryo using a 2D standing wave field. By spatially confining the microbubbles at standing wave nodes and dynamically adjusting nodal positions through acoustic frequency modulation, precise 2D positional control of microbubbles was achieved. This approach enabled programmable upstream, downstream, and cross-stream transport of microbubbles through developing vasculature. Furthermore, they developed a traveling wave-based strategy for microbubble manipulation under physiological flow conditions [64]. First, a perpendicular traveling wave field relative to the vessel wall triggered microbubble self-assembly into a microswarm through the secondary acoustic radiation force, while the primary radiation force drove collective migration toward the vessel wall. Second, activating a parallel traveling wave field along the vessel axis enabled upstream swarm navigation via acoustic radiation force.

#### Acoustic streaming

Acoustic streaming, a nonlinear acoustic phenomenon from strong wave scattering at material interfaces, enables energy transfer from the acoustic field to the hydrodynamic flow [65]. This boundary-driven streaming can be amplified through specific structures, such as sharp edges [66] and bubble cavities [67], to achieve robust propulsion (Fig. 2B). When subjected to acoustic excitation at its resonant frequencies, the structure exhibits enhanced vibration, thereby generating localized streaming vortex capable of propelling microrobot.

Microrobots propelled by bubble oscillation-induced acoustic streaming have been widely explored in acoustic fields. The strategy of propelling microrobots through oscillating bubbles was first proposed by Dijkink et al. [68]. Feng and Cho [69] used photolithography to manufacture a device propelled by bubbles, and the propulsion velocity can be up to 45 mm/s. Besides, Bertin et al. [70] designed a microrobot with an armored microbubble to extend the lifespan of the bubble. The microbubble can exist for several hours so that the microrobot can be driven stably and persistently. Furthermore, Luo and Wu [71] proposed a triangular microswimmer with 2 identical-sized bubbles to realize the turning movement. The 2 bubbles have the same diameter, while the opening diameters in contact with the fluid are different, which determines that the 2 bubbles have different resonant frequencies. Therefore, the microswimmer can be controlled to perform straight or turning movements by adjusting the ultrasonic frequency.

As another kind of specialized geometric structure that can induce local streaming by the stimulus of acoustic fields, sharp edges perform with higher robustness than fragile microbubbles. Ahmed et al. [72] proposed a microrobot based on nanowires, which is driven by the small amplitude oscillation of a propeller in standing and traveling waves. Kaynak et al. [73] presented a sperm-shaped microrobot, which can swim at a velocity of up to 1,200 μm/s using an acoustic oscillating tail in the acoustic field.

Acoustically actuated microrobots have made giant advances in their actuation mechanisms and structural design until now. However, this technology still faces challenges in biomedical applications [20]. One major limitation is the spatial resolution of acoustic field manipulation. Improving actuation precision often requires increasing the acoustic field frequency, but higher-frequency sound waves exhibit reduced penetration in deep tissues [74]. Additionally, precise directional control of acoustic microrobots remains under development, particularly in achieving 3D motion control, which often necessitates complex transducer arrays. Furthermore, acoustically actuated microrobots based on bubble oscillation are prone to failure due to bubble collapse.

### Magneto-acoustic actuation

Single magnetic and acoustic actuation of microrobots have shown their distinctive advantages, but these methods also have their inevitable limitations, as demonstrated previously. Magnetic actuation enables precise position control, but generating strong propulsion remains challenging. In contrast, low-frequency sound waves offer excellent tissue penetration and sufficient propulsion for deep-tissue microrobots but suffer from limited directional control. Additionally, while bubble collapse in acoustic fields is typically a drawback, it can be leveraged in magnetic microrobots to achieve multiple functions. As a result, researchers have increasingly focused on developing magneto-acoustic microrobots. By integrating the advantages of both magnetic and acoustic actuation, the working mechanisms of magneto-acoustic microrobots can be categorized into 2 main types: magnetic steering combined with acoustic propulsion and magnetic propulsion coupled with acoustic manipulation.

#### Magnetic steering and acoustic propulsion

**Fig. 3.**
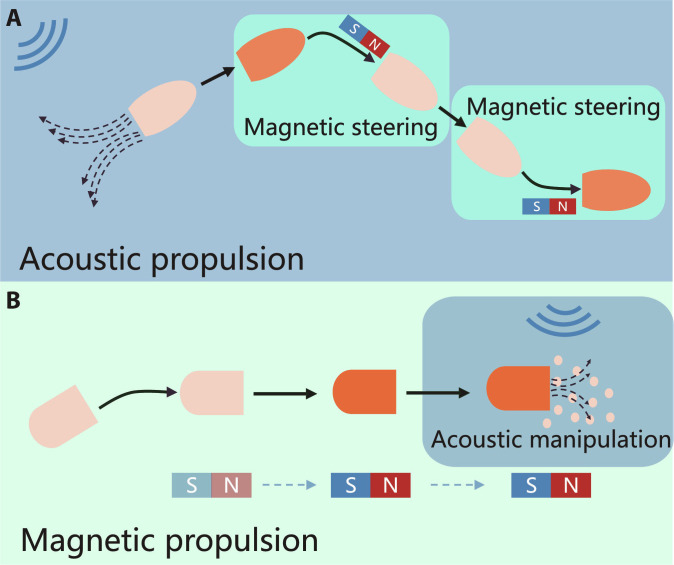
The actuation mechanisms of magneto-acoustic microrobots. (A) Microrobots are propelled by an acoustic field and steered by a magnetic field. (B) Microrobots are propelled by a magnetic field and activated by an acoustic field.

The strategy of magnetic steering and acoustic propulsion involves coupling the control of motion direction through a magnetic field with propulsion provided by an acoustic field (Fig. 3A). In hybrid magneto-acoustic actuation, the combination of magnetic steering and acoustically induced propulsion enables precise control and powerful locomotion of microrobots. Magnetic microrobots embedded with magnetic nanoparticles (MNPs) exhibit programmable directional manipulation through the alignment of induced magnetic dipoles or intrinsic magnetic moments generated by pre-magnetization with the applied magnetic field. At the same time, acoustic streaming generated by specific structures such as bubbles, asymmetric Janus structures, or cilia arrays enables efficient propulsion. By leveraging these mechanisms, microrobots can achieve controlled motion in complex environments.

**Fig. 4.**
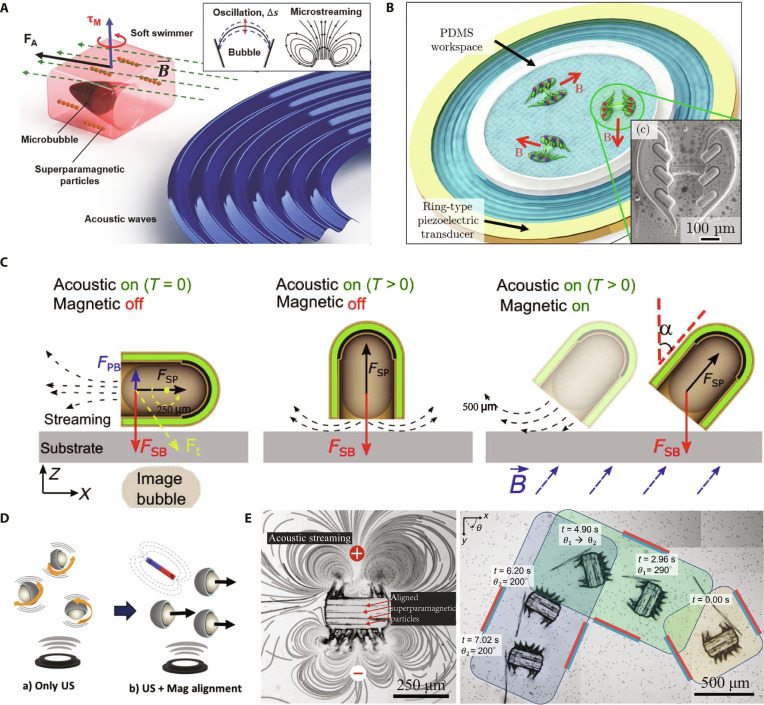
The microrobots propelled by an acoustic field and steered by a magnetic field. (A) Illustration of the soft microswimmer with a single microbubble. (Republished with permission of the John Wiley and Sons from Ref. [75]. Copyright © 2017 WILEY-VCH Verlag GmbH & Co. KGaA, Weinheim.) (B) Illustration of CeFlowBots with bubble arrays. (This figure is republished from Ref. [76] and licensed under the terms of the Creative Commons CC BY © 2021 The Authors.) (C) Illustration of the microbubble-aided microrobots. (Republished with permission of The American Association for the Advancement of Science (AAAS) from Ref. [77]. Copyright © 2019, AAAS.) (D) Illustration of the Janus micromotors. (Republished with permission of the John Wiley and Sons from Ref. [78]. Copyright © 2020 Wiley-VCH GmbH.) (E) The starfish-inspired microrobot with an asymmetric cilia array. (This figure is republished from Ref. [79] and licensed under the terms of the Creative Commons CC BY 3.0 © 2024 The Authors.)

Magnetically guided robots propelled by bubble-induced streaming are continuously evolving. Ahmed et al. [75] reported a microrobot with a microcavity structure embedded with superparamagnetic nanoparticles. The bubble trapped in the microcavity oscillates under acoustic stimulation to generate substantial propulsive force, while the magnetically responsive particles form reconfigurable chains that enable steering by external magnetic fields (Fig. 4A). Besides, the arrayed design of bubbles enhances acoustic streaming force, enabling more efficient propulsion. Mohanty et al. [76] introduced CeFlowBot, which features an array of 6 entrapped air bubbles within its body, which vibrate under the excitation of sound waves to generate a directed flow through its internal channel. Additionally, magnetic layers are incorporated into its design, enabling it to be controlled under a uniform magnetic field and simultaneously propelled using an acoustic field. By harnessing this hybrid magneto-acoustic propulsion system, they demonstrated the ability of CeFlowBot to follow complex trajectories, showcasing its versatile movement capabilities (Fig. 4B). Furthermore, Ren et al. [77] presented another method of bubble propulsion combined with magnetic steering to realize 3D movement of the microrobot. Under acoustic field activation alone, the microrobot remains vertically oriented on the substrate surface due to the secondary Bjerknes force between the bubble and the substrate. When both the magnetic and acoustic fields are activated, the microrobot’s orientation relative to the substrate changes, enabling acoustic streaming to provide propulsion (Fig. 4C). Besides bubbles, other asymmetric structures have also been designed to propel microrobots. Valdez-Garduño et al. [78] demonstrated a novel motion control strategy for Janus micromotors based on magneto-acoustic hybrid actuation. The Janus microstructure, composed of silica microspheres with hemispherical high-density coatings, generates propulsion through the asymmetric oscillation-induced streaming flow under ultrasound excitation. By employing an external magnetic field to fix the micromotor orientation, this system achieves directional propulsion (Fig. 4D). Dillinger et al. [79] developed a soft microrobot with the asymmetric cilia array, where pre-aligned MNPs embedded during fabrication enable directional control by aligning with an external magnetic field. Acoustic-responsive polymeric cilia generate propulsion force through high-frequency, low-amplitude vibrations. This design eliminated bubble-dependent mechanisms for enhanced operational stability (Fig. 4E).

#### Magnetic propulsion and acoustic manipulation

The strategy of magnetic propulsion and acoustic manipulation leverages dual-field synergy: Magnetic field enables targeted locomotion to the desired location, while acoustic field triggers on-demand functional activation (Fig. 3B). While magnetic fields have enabled reliable actuation and precise positioning of microrobots, executing complex manipulations requires complex deformation of microrobot and stringent external magnetic field requirements [80,81]. To overcome these limitations, researchers have integrated acoustic field activation as a complementary functionalization strategy. Under acoustic excitation, on-demand functionalization (e.g., fluid mixing and drug release) is realized by the effects of acoustic streaming, cavitation, and sonochemical reactions.

**Fig. 5.**
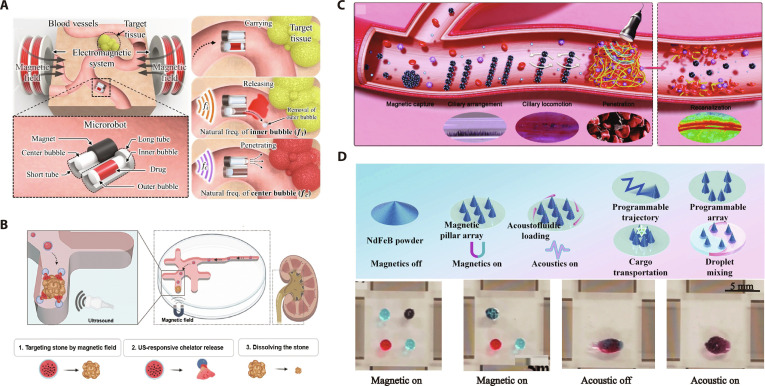
The microrobots propelled by a magnetic field and conducting manipulation by an acoustic field. (A) Illustration of the microrobot with 2 tubes guided by a magnetic field and delivering drug by oscillating bubbles. (Republished with permission of Elsevier from Ref. [82]. Copyright © 2020 Elsevier B.V.) (B) Illustration for magnetic delivery of microcapsules containing a chelator via ultrasound-responsive release. (Republished with permission of Royal Society of Chemistry from Ref. [34]. Copyright © 2023 Royal Society of Chemistry.) (C) Illustration of enhanced penetration of drugs by cilia-mimic locomotion of magnetic colloidal collectives combined with low-intensity ultrasound. (This figure is republished from Ref. [83] and licensed under the terms of the Creative Commons CC BY 4.0 © 2024 The Authors.) (D) Magnetic micropillars for droplet manipulation via acoustic activation. (Republished with permission of Elsevier from Ref. [84]. Copyright © 2024 Elsevier B.V.)

The multifunctionality of magnetically actuated microrobots under acoustic fields is continuously being explored and developed. Jeong et al. [82] proposed a microrobot with 2 microtubes of different lengths and a small permanent magnet. Under the guidance of a magnetic field, the robot navigates to the targeted location, where acoustic activation of bubbles is employed. First, the oscillation of the inner bubble inside the long tube is triggered to release the drug encapsulated by the outer bubble. Then, unidirectional microstreaming generated by the bubble in the short tube directs the drug for targeted penetration into the tissue (Fig. 5A). Besides, Kaang et al. [34] developed a delivery method for magnetic microcapsules composed of a poly (lactic-co-glycolic acid) (PLGA) shell encapsulating MNPs and therapeutic agents. Guided by the external magnetic field, the microcapsules navigate to the target therapeutic region. Upon ultrasound stimulation, cavitation-induced shell rupture triggers the release of encapsulated drugs, achieving on-demand payload delivery (Fig. 5B). Wu et al. [83] developed a strategy to enhance thrombolytic drug penetration by modulating the cilia-like motion of magnetic microspheres under a torque–force vortex magnetic field with low-intensity ultrasound activation. The vortex field aligns the collectives perpendicular to the rotation plane for vortex motion, while ultrasound enhances their penetration and diffusion within the thrombus and the enzyme activity to improve thrombolytic efficacy (Fig. 5C). Furthermore, Pan et al. [84] integrated magnetic pillar arrays with acoustic streaming to achieve multifunctional manipulation. NdFeB particles self-assemble into reconfigurable magnetic micropillar arrays through the magnetic field gradient. Under acoustic excitation, these pillars generate localized acoustic streaming vortices capable of particle trapping or fluid perturbation. Programmable magnetic field modulation enables precise cargo transport and droplet mixing through dynamic pillar reconfiguration (Fig. 5D).

The above examples demonstrate the synergistic advantages of integrating acoustic and magnetic actuation in microrobotics. A magnetic field is typically anisotropic; that is, the direction of the lines of magnetic susceptibility always points from the N-pole to the S-pole, and there tends to be a stronger magnetic gradient in the part nearer to the pole. This means that magnetic gradients and magnetic inductance can provide anisotropic and accurate directional control for robotic manipulation, but it is difficult to provide uniform and robust velocity control. The acoustic field, conversely, is characterized as an evanescent field, wherein microrobotic particles exhibit a nonselective response to acoustic forces across different directions—a phenomenon rooted in their inherent isotropy. This characteristic implies that the superposition of multiple acoustic sources, coupled with reflection and scattering effects during wave propagation, collectively modulates the trajectory and orientation of the particles, thereby influencing their directional controllability. In the integration of magnetic steering and acoustic propulsion, the acoustic field bears the energy consumption for rapid propulsion, while the magnetic field provides only the energy needed for directional adjustment, thereby overcoming the limitation of rapid magnetic field attenuation and optimizing energy efficiency. Combining magnetic propulsion and acoustic manipulation enables decoupled control of locomotion and functional operations, preventing mutual interference between propulsion and manipulation tasks in single-field systems. This dual-field decoupling reduces the complexity of external field actuation while enabling multifunctional capabilities. Overall, the acoustic and magnetic hybrid actuation improves the performance of the microrobot and can realize more diversified functions. A comparison of magnetic, acoustic, and magneto-acoustic microrobots is presented in Table 1.

**Table 1.** Comparison of magnetic, acoustic, and magneto-acoustic microrobots

Type of microrobot	Speed/(μm·s^−1^)	Directional control	Demonstrated functions	References
Magnetic microrobot	7.5–600	Anisotropic	Particle manipulation, droplet manipulation, cell manipulation, cargo transport, drug delivery,	[47,48,50,53–55]
Acoustic microrobot	~100–10,000	Isotropic	Particle manipulation, cell manipulation, cargo transport, drug delivery	[63,64,72–75,97–99]
Magneto-acoustic microrobot	250–2,600	Anisotropic	Particle manipulation, droplet manipulation, cell manipulation, cargo transport, drug delivery, enhanced drug absorption, imaging	[34,49,76,77,79,82–84]

## Biomedical Applications of Magneto-Acoustic Microrobots

In recent years, magneto-acoustic microrobots have rapidly advanced in the biomedical field due to their robust propulsion, precise positioning, multifunctionality, good biocompatibility, and remote controllability. The solution of magneto-acoustic actuation has been increasingly adopted to solve problems in biomedical and diagnostic applications, especially in the following 3 fields: targeted drug delivery, minimally invasive surgery, and medical imaging.

### Targeted drug delivery

Targeted drug delivery is vital in biomedicine as it not only enhances therapeutic efficacy but also reduces systemic side effects [85]. However, challenges such as drug instability, precise localization, and penetration of biological barriers hinder traditional passive transport approaches [86]. Magneto-acoustic microrobots offer unique advantages for drug delivery: Their small size allows them to navigate complex biological environments and external acoustic and magnetic fields, enabling precise guidance and control. Garcia-Gradilla et al. [87] proposed an acoustic propulsion, magnetically guided nanowire motor with a diameter of 0.25 μm and a length of 1.8 μm. Its capability for drug loading and pH-responsive release was successfully demonstrated in vitro. This nanomotor demonstrates efficient propulsion capabilities in physiological environments such as serum and saliva while achieving precise navigation along complex trajectories under magnetic guidance.

**Fig. 6.**
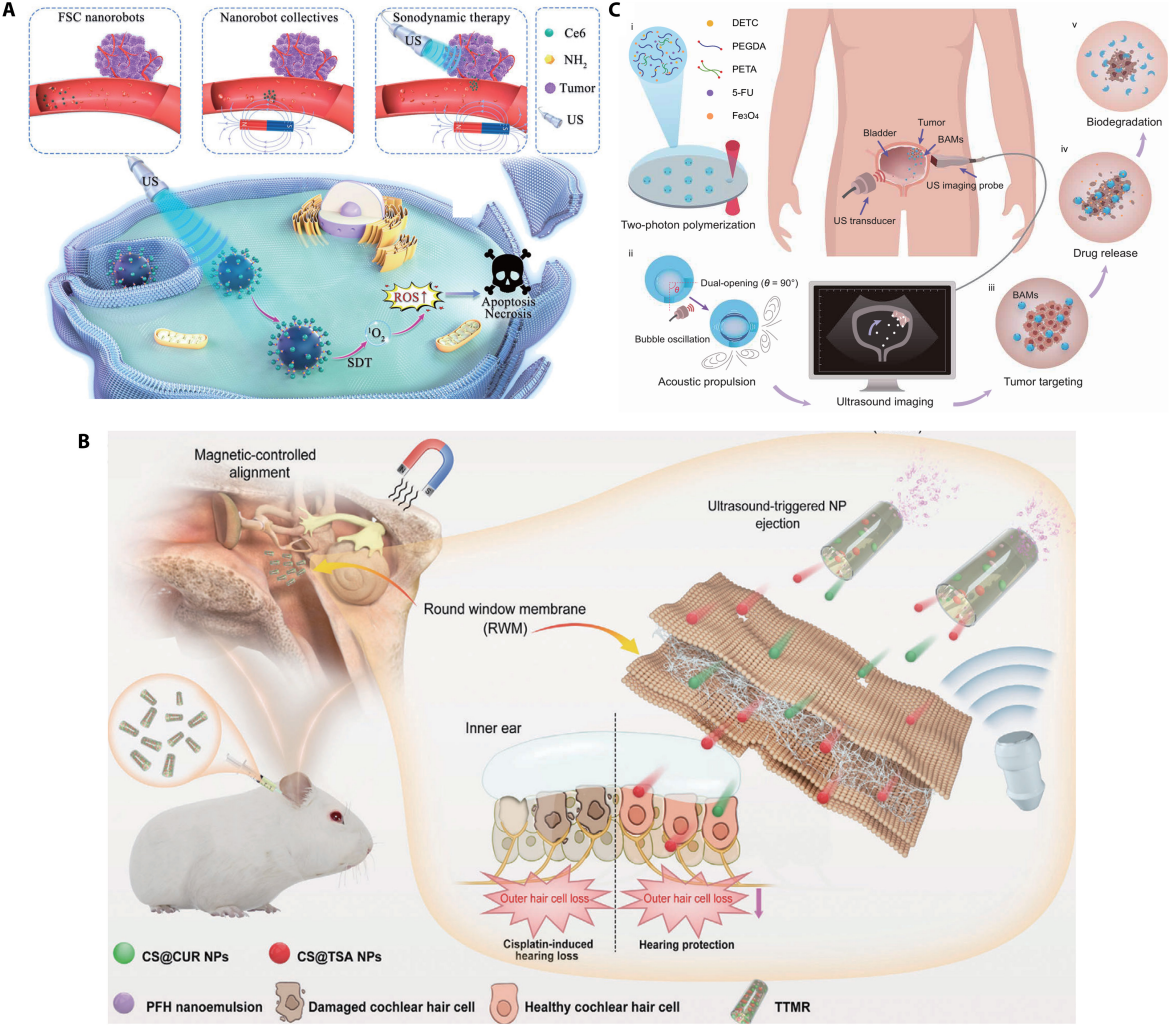
The magneto-acoustic microrobots for drug delivery. (A) Illustration of the magnetically actuated sonodynamic nanorobot. (This figure is republished from Ref. [35] and licensed under the terms of the Creative Commons CC BY 4.0 © 2024 The Authors.) (B) Illustration of the tube-type microrobot penetrating the round window membrane for on-demand multidrug delivery. (Republished with permission of the John Wiley and Sons from Ref. [89]. Copyright © 2024 Wiley-VCH GmbH.) (C) Illustration of the hydrogel microrobot BAMs for drug release in vivo. (Republished from Ref. [33]. Copyright © 2014, AAAS.)

Moreover, local physical effects (e.g., local flow field induced by acoustic stimulus) can be exploited for targeted drug release, resulting in a high concentration of the therapeutic agent at the target location and thus improved treatment outcomes. Zhou et al. [35] introduced a magnetically actuated sonodynamic nanorobot system for targeted tumor therapy. Combining magnetic cores with the sonosensitizer, the nanorobots are magnetically guided to aggregate and navigate to tumor sites, enabling precise delivery of sonosensitizer. This synergy of magnetic actuation and sonodynamic therapy amplifies reactive oxygen species (ROS) generation, enhancing cytotoxicity on tumor cells (Fig. 6A). Chertok and Langer [88] developed magnetic microbubbles with strong magnetic and acoustic properties. The magnetic microbubbles are fabricated from magnetic composites with drugs attached to their surfaces. Under the influence of a magnetic field, these microbubbles can circulate in the body, avoid deposition in the capillaries, and accumulate at the lesion sites. Subsequently, focused ultrasound is used to rupture the microbubbles, triggering drug release. Yi et al. [89] proposed a novel tube-type microrobot penetrating the round window membrane for on-demand multidrug delivery. Constructed via template-assisted layer-by-layer assembly of chitosan/Fe₃O₄/SiO₂, it loads anti-ototoxic agents along with perfluorohexane (PFH). The microrobots are actuated under the external magnetic field and utilize ultrasound-triggered PFH vaporization to propel the drugs into the inner ear. Drug delivery efficiency is precisely tunable by adjusting formulation ratios, demonstrating a controllable approach for overcoming biological barriers in targeted therapy (Fig. 6B).

With continuous technological advances, magneto-acoustic microrobots hold great promise for providing more efficient and safer drug delivery solutions in precision medicine. The bioresorbable acoustic microrobots (BAMs) composed of hydrogel, MNPs, and therapeutic drugs have been proposed. When the acoustically driven and magnetically guided robot reaches the tumor site in mice, the drug is released as the robot degrades. They are also capable of continuous directional propulsion in various biological fluids. The hydrophilic hydrogel shell not only mitigates the aggregation of BAMs in vivo but also facilitates their rapid degradation, ensuring enhanced safety for in vivo drug delivery [33] (Fig. 6C). Magneto-acoustic microrobots are also integrated with cellular components to improve biocompatibility. Wu et al. [90] reported a microrobot based on red blood cells (RBCs). The anti-cancer drug doxorubicin and MNPs were encapsulated in the RBC microrobot. Under acoustic propulsion and magnetic steering, the RBC microrobot can load and transport drugs.

### Minimally invasive surgery

Magneto-acoustic microrobots can be engineered as microsurgical tools capable of directly penetrating cellular and tissue barriers, offering promising solutions for minimally invasive procedures such as tissue puncture, biofilm degradation, and thrombus removal. Owing to their miniature size, superior maneuverability, and capability for remote control, magneto-acoustic microrobots are capable of accessing lesion sites that remain unreachable by conventional surgical instruments such as catheters or scalpels. Upon arrival at the target region, acoustic stimulation induces localized dynamic responses that enhance tissue penetration. In addition, magneto-acoustic microrobots can reduce incision size, minimize tissue damage and infection risk, and shorten patient recovery time.

**Fig. 7.**
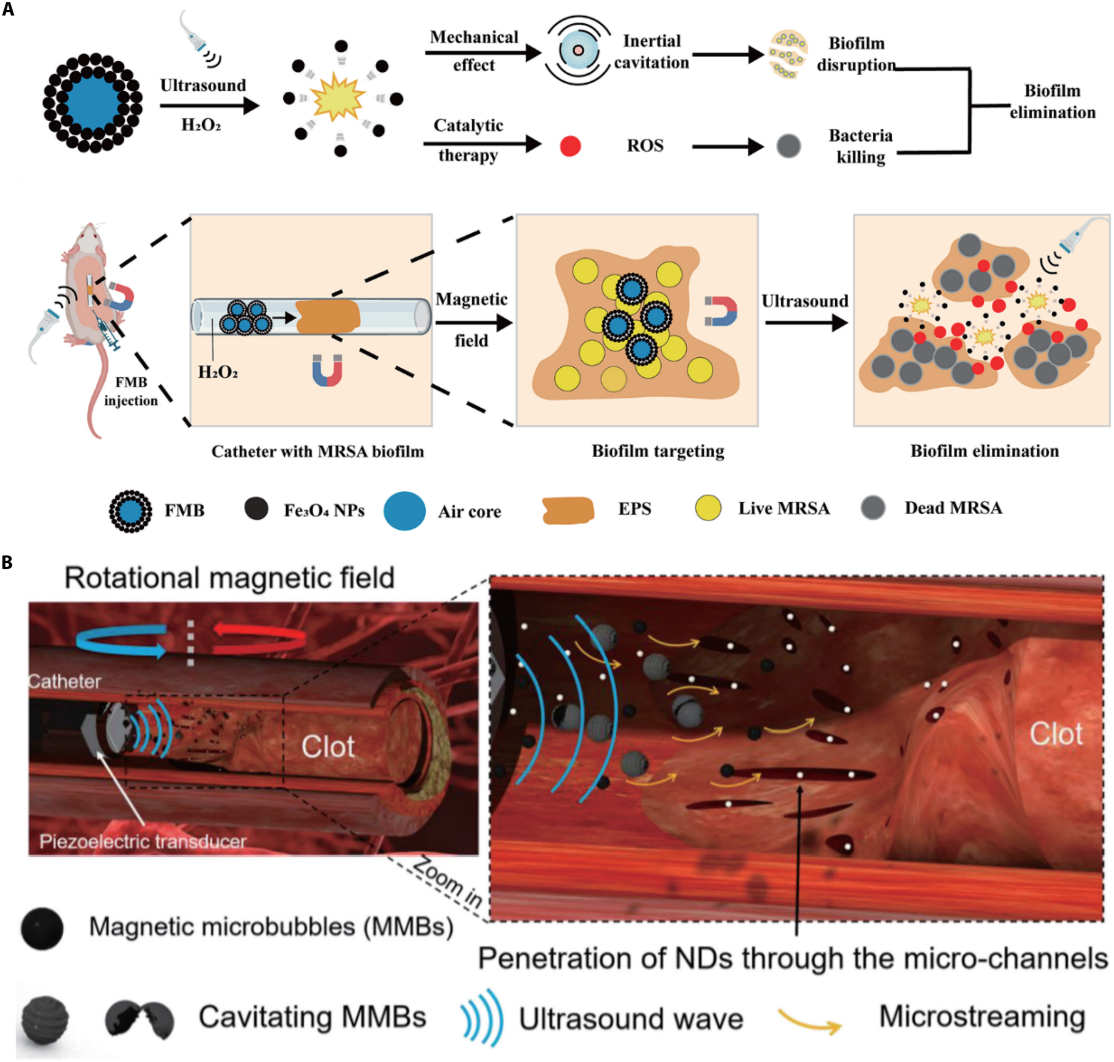
The magneto-acoustic microrobots for minimally invasive surgery. (A) Illustration of the ultrasound-responsive magnetic microbubble for methicillin-resistant *S. aureus* biofilm eradication. (This figure is republished from Ref. [91] and licensed under the terms of the Creative Commons CC BY 4.0 © 2024 The Authors.) (B) Illustration of the ultrasound-responsive magnetic microbubble system designed to enhance thrombolytic efficiency. (Republished with permission of Elsevier from Ref. [92]. Copyright © 2021 Elsevier B.V.)

Lighthill [65] reported a microbullet that can be rapidly propelled by an acoustic field and guided by the magnetic field, representing an efficient propulsion technique for tissue puncture. Under ultrasound stimulation, the perfluorocarbon droplet vaporizes rapidly to generate a large enough propulsion force to drive the robot to penetrate and dissect sheep kidney tissue. Lu et al. [91] developed an ultrasound-responsive magnetic microbubble for methicillin-resistant *Staphylococcus aureus* biofilm eradication. Guided by a magnetic field, microbubbles target and accumulate at the biofilm site. Under ultrasound stimulation, the cavitation-induced mechanical force disrupts the biofilm, while the released Fe₃O₄ nanoparticles penetrate the biofilm and catalyze H₂O₂ to generate ROS, effectively killing bacteria (Fig. 7A). Zhang et al. [92] introduced an ultrasound-responsive magnetic microbubble system designed to enhance thrombolytic efficiency. The microbubbles’ cavitation and rotation induce microstreaming that mechanically damages the thrombus’s fibrin network, forming microchannels on its surface. These channels then allow drug-loaded nanodroplets to penetrate, where cavitation effects within the fibrin network ultimately lead to thrombus rupture (Fig. 7B).

### Medical imaging

**Fig. 8.**
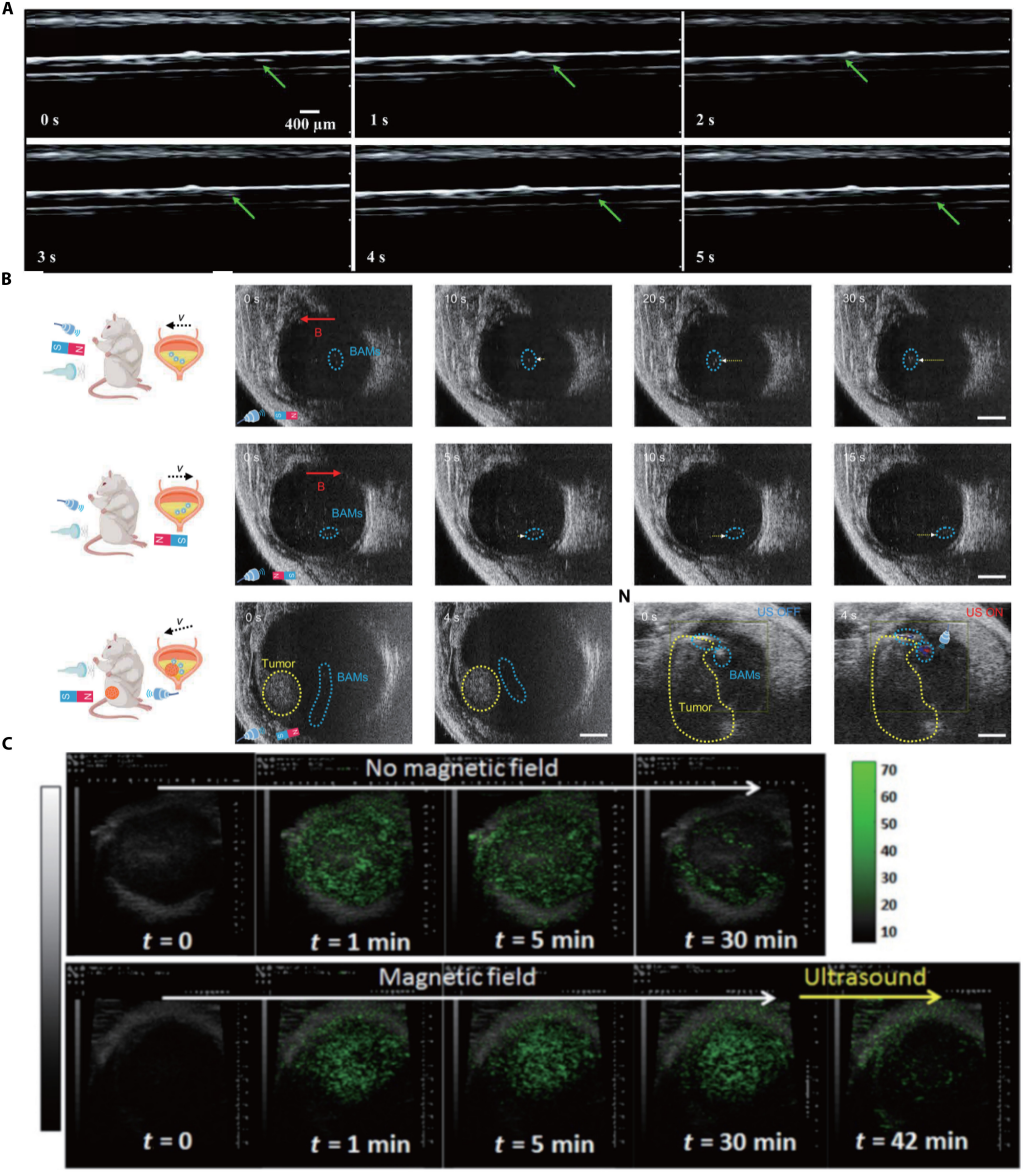
The magneto-acoustic microrobots for imaging. (A) Representative ultrasonograms of the tumor at different time points. (This figure is republished from Ref. [93] and licensed under the terms of the Creative Commons CC BY-NC 4.0 © 2020 The Authors.) (B) Ultrasound imaging of IRONSperm clusters (green arrow) within medium flow streams. (Republished from Ref. [33]. Copyright © 2014, AAAS.) (C) The corresponding ultrasound images of propulsion of BAMs with magnetic guidance in the bladder of a mouse. (This figure is republished [88] and licensed under the terms of the Creative Commons CC BY-NC 4.0 © 2018 The Authors.) The scale bar in (B) represents 2 mm.

Microrobots play a crucial role in clinical settings through individual or collective monitoring. Advanced medical microrobots can be dynamically tracked in vivo using real-time imaging technologies to ensure precise navigation to targeted regions. Magneto-acoustic microrobots, engineered with specialized materials such as gas-filled microbubbles or MNP coatings, enhance acoustic contrast, particularly for deep-tissue visualization, thereby improving resolution in ultrasound-guided biomedical imaging. This capability enables real-time monitoring of microrobots’ movement and localization with high spatiotemporal resolution, offering a robust tool for tracking dynamic biological processes in vivo. Magdanz et al. [93] developed a sperm-templated microrobot composed of a bovine sperm cell coated with iron oxide nanoparticles. The nanoparticle coating enables magnetic field actuation and increases the acoustic impedance of the sperm cells, enhancing the echogenicity of the IRONSperm clusters. This design allows real-time localization through ultrasound feedback, demonstrating a hybrid biohybrid system for targeted actuation and imaging-guided navigation (Fig. 8A). The aforementioned BAMs possess a dual-opening bubble cavity, with internally trapped bubbles serving as effective contrast agents to enhance the high-resolution imaging of BAMs in the mouse bladder [33] (Fig. 8B).

Moreover, the aggregation of microrobots at lesion sites enhances imaging resolution by amplifying signal contrast via localized high-concentration imaging agents. Chertok and Langer [88] developed magnetic microbubbles capable of ultrasonographic monitoring, magnetic targeting-driven aggregation within tumor vasculature, and on-demand drug release via collapse under focused ultrasound. These microbubbles exhibit acoustic sensitivity comparable to clinically used contrast agents, enabling real-time localization through ultrasound imaging monitoring. As shown in Fig. 8C, after intravenous injection of magnetic microbubbles in mice, ultrasound contrast enhancement was observed at the tumor site. Compared to scenarios without magnetic field application, the microbubbles demonstrated magnetic field-enhanced accumulation at the target region and rapid collapse upon ultrasound application. This work [88] further examined the in vivo distribution of magnetic microbubbles in mice using fluorescence imaging. As mentioned earlier, magnetic microbubbles can achieve high abundance locally under the action of focused ultrasound. Therefore, microbubbles containing fluorescent substances can also accumulate in tumors, thereby enhancing the effectiveness of fluorescence measurements. This design demonstrates the potential of combining ultrasound monitoring with fluorescence or x-ray imaging techniques to achieve enhanced and multidimensional in vivo imaging.

## Conclusions and Outlook

Microrobots driven by magnetic and acoustic fields have become powerful tools in biomedical applications due to their excellent biocompatibility and tissue penetration. However, single physical actuation methods face the challenges of limited propulsion of the magnetic actuation and difficult direction control of the acoustic actuation. Recent advances in magneto-acoustic microrobots have shown alternative solutions to these challenges. This review summarizes the actuation mechanisms of magnetic, acoustic, and magneto-acoustic microrobots, highlighting the advantages of magneto-acoustic microrobots in biomedicine. Magnetic and acoustic actuation offers complementary advantages in microrobotic control. Magnetic actuation excels in directional control, allowing for high-precision trajectory guidance and remote manipulation. On the other hand, acoustic actuation provides strong propulsion and deep tissue penetration and enables functional activation through physical effects such as acoustic streaming, cavitation, and sonochemical reactions. The integration of these 2 actuation mechanisms combines the efficient propulsion of acoustic fields with the precise navigation enabled by magnetic fields, where the magnetic field ensures directional accuracy, while the acoustic field primarily drives locomotion. Moreover, the physical effects induced by acoustic excitation facilitate microscale operations, thereby extending the functional capabilities of magnetic microrobots and supporting their application in complex physiological environments. In recent years, magneto-acoustic microrobots have overcome the limitations associated with single-field actuation, offering high propulsion forces, precise positioning, low energy consumption, decoupled control of locomotion and multifunctionality, and excellent biocompatibility. These features enable them to perform essential biomedical tasks such as drug transport. Moreover, they can serve as surgical tools for tissue puncture and biofilm degradation, and as imaging agents for both in vitro and in vivo applications using ultrasound, magnetic, and other modalities. Additionally, magnetic-acoustic robots offer potential advantages in microgravity environments and space life science due to their versatility and integrated design [94–96].

Magneto-acoustic microrobots hold great promise for biomedical applications, but several key challenges must be addressed [8,26–28]. Ensuring in vivo safety and biocompatibility is critical, as long-term accumulation of MNPs after microrobot degradation may pose health risks, despite progress in using biodegradable materials and incorporating MNPs into robot structures. Clinical translation also demands reliable operation in complex and dynamic physiological environments. However, current control strategies, which have been primarily validated in vitro, exhibit limited adaptability. Furthermore, while swarm-based operation enhances efficiency at the microscale, achieving individual control within swarms remains difficult. Accordingly, to promote the practical development of magneto-acoustic microrobots, future advances could focus on the following 3 key directions. (a) New magnetic materials with low toxicity, good biocompatibility, and controllable biodegradability should be developed. In addition, strategies for removing residual magnetic particles after the robots degrade should be explored, such as guiding them to excretory organs using external magnetic fields and relying on natural metabolic pathways for removal; (b) Magneto-acoustic control should be integrated with real-time, high-resolution medical imaging technologies to develop artificial intelligence-based control algorithms guided by image feedback. Model predictive control (MPC) can be employed to build microrobot motion models that account for environmental disturbances and predict optimized inputs. Reinforcement learning can be used to enable autonomous adaptation within in vivo environments. Ultimately, by dynamically adjusting the parameters of the magnetic and acoustic fields, the system can compensate in real time for internal disturbances such as blood flow; (c) In the design of microrobot swarms, individual robots can be endowed with distinct physical characteristics, such as differences in shape, size, or material composition. These variations can result in different acoustic resonance frequencies or magnetic response thresholds, enabling the switching between collective control and individual control modes. Overcoming these obstacles will enable magneto-acoustic microrobots to become powerful tools in precision medicine, substantially advancing modern biomedicine.

## Data Availability

The processed data are available from the corresponding author upon reasonable request.
